# Frictional force analysis of stent retriever devices using a realistic vascular model: Pilot study

**DOI:** 10.3389/fneur.2022.964354

**Published:** 2022-08-24

**Authors:** Youngseok Kwak, Wonsoo Son, Byoung-Joon Kim, Myungsoo Kim, Sang-Youl Yoon, Jaechan Park, Jongkyeong Lim, Joonwon Kim, Dong-Hun Kang

**Affiliations:** ^1^Department of Neurosurgery, School of Medicine, Kyungpook National University, Daegu, South Korea; ^2^Department of Mechanical Engineering, Gachon University, Seongnam-si, South Korea; ^3^Department of Mechanical Engineering, Pohang University of Science and Technology (POSTECH), Pohang, South Korea; ^4^Departement of Radiology, School of Medicine, Kyungpook National University, Daegu, South Korea

**Keywords:** acute ischemic stroke, vascular model, frictional retrieval force, Trevo XP, Solitaire 2, Eric 4

## Abstract

**Objective:**

To date, no vascular model to analyze frictional forces between stent retriever devices and vessel walls has been designed to be similar to the real human vasculature. We developed a novel *in vitro* intracranial cerebrovascular model and analyzed frictional forces of three stent retriever devices.

**Methods:**

A vascular mold was created based on digital subtraction angiography of a patient's cerebral vessels. The vascular model was constructed using polydimethylsiloxane (PDMS, Dow Corning, Inc.) as a silicone elastomer. The vascular model was coated on its inner surface with a lubricating layer to create a low coefficient of friction (~0.037) to closely approximate the intima. A pulsatile blood pump was used to produce blood flow inside the model to approximate real vascular conditions. The frictional forces of Trevo XP, Solitaire 2, and Eric 4 were analyzed for initial and maximal friction retrieval forces using this vascular model. The total pulling energy generated during the 3 cm movement was also obtained.

**Results:**

Results for initial retrieval force were as follows: Trevo, 0.09 ± 0.04 N; Solitaire, 0.25 ± 0.07 N; and Eric, 0.33 ± 0.21 N. Results for maximal retrieval force were as follows: Trevo, 0.36 ± 0.07 N; Solitaire, 0.54 ± 0.06 N; and Eric, 0.80 ± 0.13 N. Total pulling energy (N·cm) was 0.40 ± 0.10 in Trevo, 0.65 ± 0.10 in Solitaire, and 0.87 ± 0.14 in Eric, respectively.

**Conclusions:**

Using a realistic vascular model, different stent retriever devices were shown to have statistically different frictional forces. Future studies using a realistic vascular model are warranted to assess SRT devices.

## Introduction

Acute ischemic stroke (AIS) is one of the leading causes of mortality in the world and is associated with a high rate of morbidity (1). Despite initial poor outcomes for stent retriever thrombectomy (SRT) in three early trials in 2013 (2–4), five key randomized controlled studies in 2015 (5–9) demonstrated good outcomes for SRT relative to controls. As a consequence of these successful trials and later validation studies, SRT is now considered the gold standard technique for patients with large vessel occlusion AIS (10).

Although SRT is an established technique, it still carries a risk of complications such as subarachnoid hemorrhage and arterial dissection (11). During retrieval, frictional resistance can stretch vessels and cause dissection or rupture (12, 13). Theoretically, different SRT devices, such as Trevo, Solitaire, and Eric, exert different frictional forces on vascular architecture, which could lead to different rates of associated complications, but evidence on this is still limited. Animal models have provided some evidence on this, but the implications of frictional force effects of SRT devices in humans are limited due to differences in vessel size and curvature (14). Artificial vascular models have been introduced to explore these issues. There have been several experimental models with a shape similar to that of the human vasculature, although they did not experiment on frictional force in stent retrievers (15–18). However, these models did not apply a human-like inner surface, which is not suitable for measuring the friction force of a stent. To date, there has been no vascular model to analyze interaction forces between stent retriever devices and artificial vessel walls that is designed to be similar to the real human vasculature.

We developed a novel *in vitro* intracranial cerebrovascular model designed to quantitatively estimate friction forces of stent retriever devices. In this pilot study, we compared the friction forces for three commercially available stent retriever devices, the Trevo XP, the Solitaire 2, and the Eric 4.

## Materials and methods

This study received approval from our local institutional review board.

### Preparation of vascular mold for artificial vascular model

**Figure 1:**
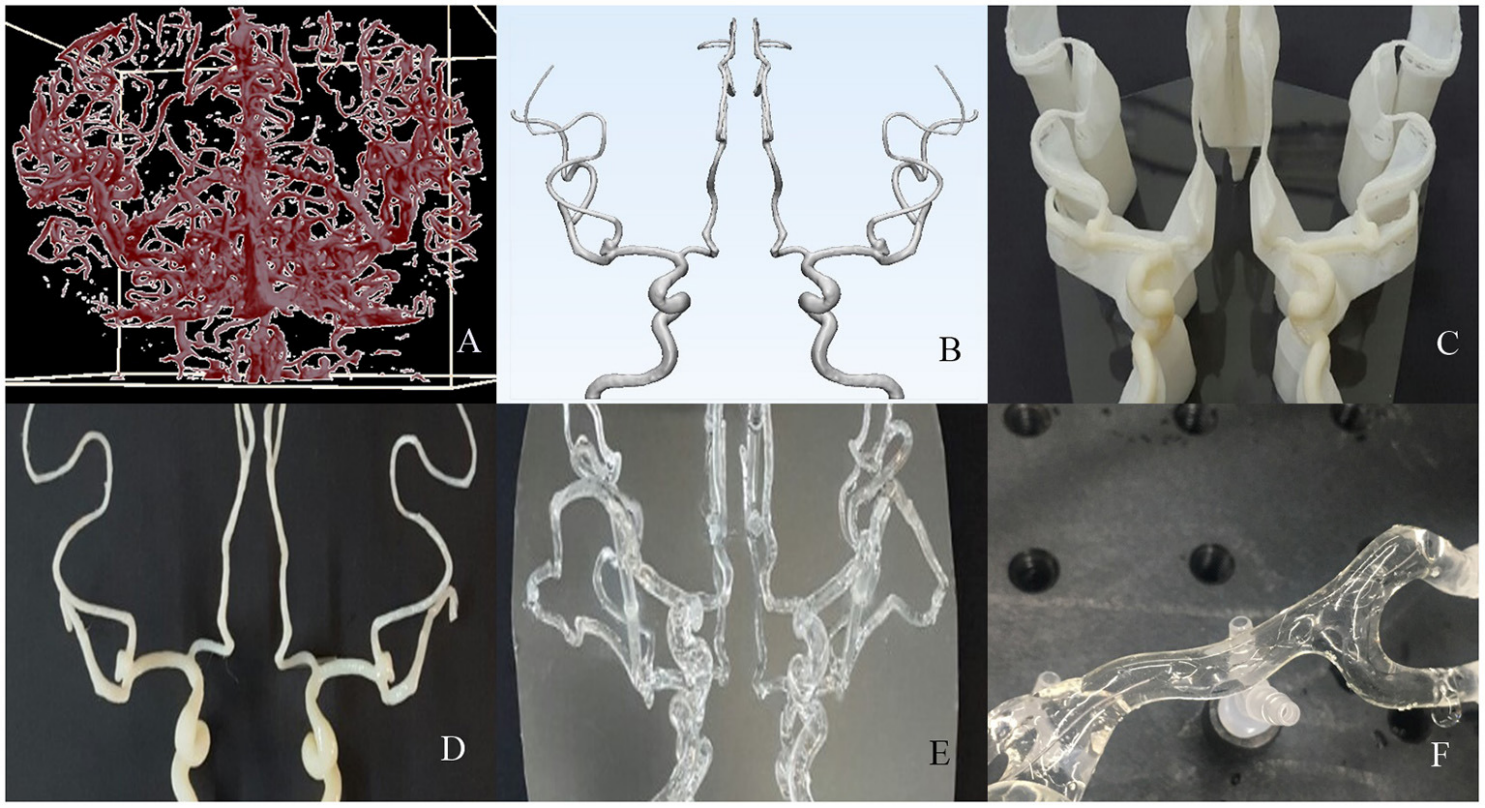
The process of creating an artificial vascular model. **(A,B)** Using a source image of 3-dimensional rotational digital subtraction angiography, format the design of the vascular model suitable for 3d printing. **(C,D)** The mold was printed with an acrylonitrile butadiene styrene resin and smoothened with acetone. **(E)** A transparent and hollow vascular model was made by dip-coating methods with silicone elastomer. **(F)** After a lubricating coating was applied on the inner surface, it was finally applied to the stent retrieval experiment.

To prepare the model, a patient's cerebral blood vessels were visualized using digital subtraction angiography (DSA), and the vascular model was constructed based on the images. First, vascular images were formatted with a virtual reality modeling language (VRML) file. Once acquired, the images were imported into Mimics^®^ software (Materialize, Inc.) and converted into a stereolithography (STL) file (Figure 1A). The STL file format was then imported into Meshmixer^®^ software (Autodesk, Inc.). Following that, the shape, size, and configuration of the vascular model were modified to the desired shape (Figure 1B). A standardized shape of the cerebral vessel was molded from the proximal internal carotid artery to the M2 segment of the middle cerebral artery. Finally, the 3D substrate was printed with an acrylonitrile butadiene styrene (ABS) resin utilizing a fused deposition method (FDM) 3D printer (uPrint^®^ SE Plus, Staratasys, Inc.) (Figure 1C). Then, the vascular 3D substratemade of ABS resin was smoothened with acetone for 30 s (Figure 1D).

### Fabrication of artificial vascular model

The artificial vascular model was constructed using a replica molding technique with the printed vascular 3D substrate. First, the vascular model was dip-coated three times with a silicone elastomer. Polydimethylsiloxane (PDMS, Dow Corning, Inc.) as a silicone elastomer was thoroughly mixed in a plastic beaker at a 10:1 weight ratio of base and curing agent, respectively. For one-time dipping, a one-time curing process was performed. Before curing, the model was air-dried for 10 min to form an even thickness. The attainable membrane thickness was controlled to determine the withdrawing velocity. The withdrawing velocity was precisely adjusted using a dip coating system consisting of a Z-axis motorized stage and a programmable motion controller. The membranes dip-coated with PDMS were cured in an 80°C oven for 40 min. Finally, a hollow artificial vascular model composed of only PDMS film was obtained by dissolving the mold in acetone with sonication for 2 h (Figure 1E). The PDMS, which is stable in transparent and diverse environments, acts as a media with an elastic modulus (~3 MPa) similar to that of the original blood vessel (19).

### Lubricated coatings inside artificial vascular model

The artificial vascular model was coated on its inner surface with a lubricated layer to satisfy all the conditions necessary for stent retriever simulation. For an even and thin lubricant coating of the inner surface of the tortuous vascular model, we used a coating method of polymerizing a hydrogel pre-gel solution (10% acrylamide in DI water, containing hydrophilic photocurable initiators [1% Irgacure 2959]) with ultraviolet (365 nm) light (20). A more detailed fabrication process and inner surface coating method of our vascular model were introduced in previously published papers (20, 21). The lubricated layer was determined to have a low coefficient of friction (~0.037) similar to that of native tissues to closely approximate the intima (22). Additionally, this provided an environment for pulsation using blood-like liquids and external stimuli during the stent retrieval test (Figure 1F).

### Experimental setup to analyze pulling load of stent retrievers

**Figure 2:**
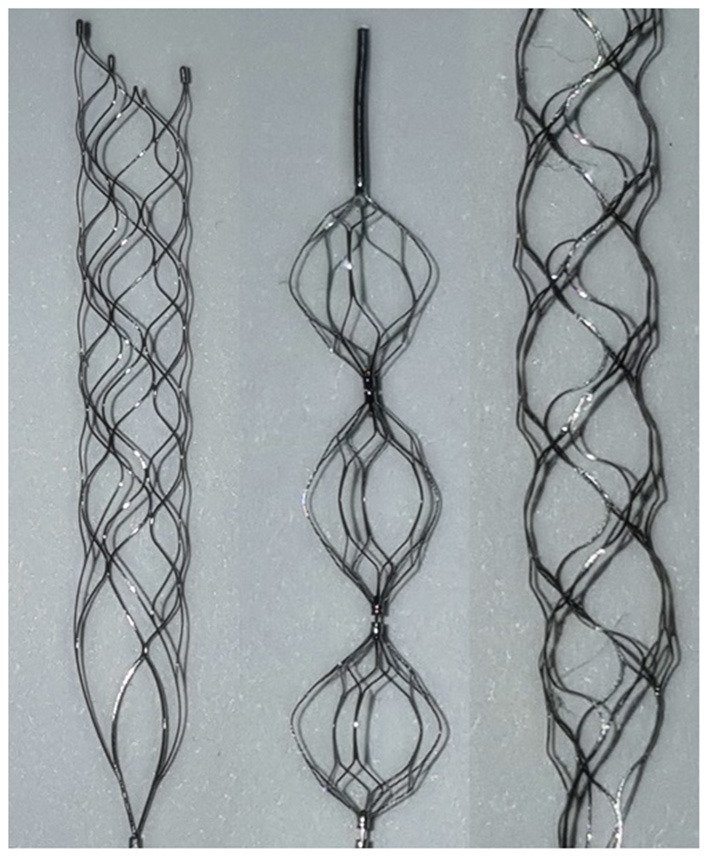
Stent types used in this experiment: Solitaire 2 (left), Eric 4 (middle), Trevo XP (right). Solitaire and Trevo are cylindrical, while Eric is a series of spheres.

**Figure 3:**
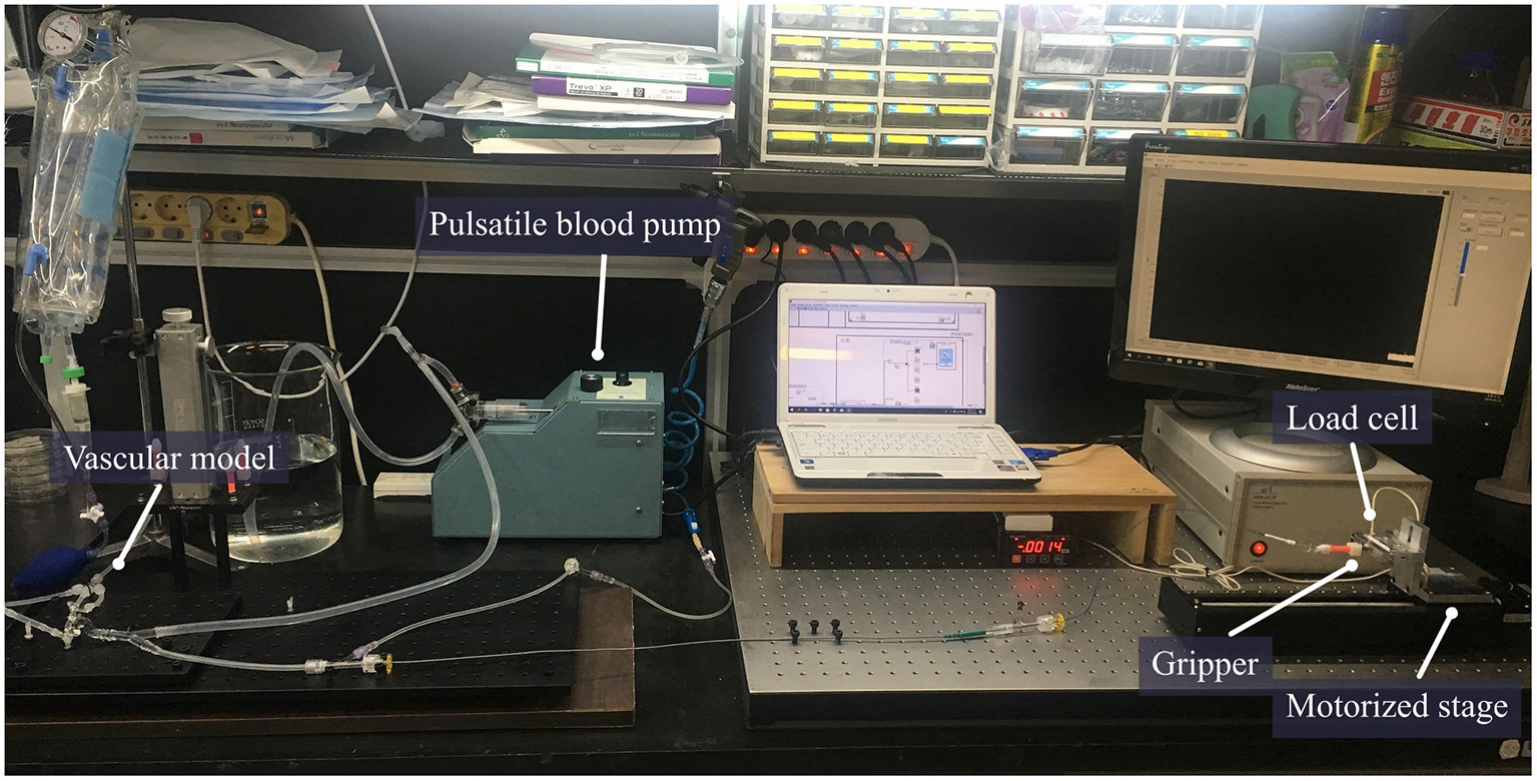
The setting for measurement of stent retrieval frictional force. A circulation system including a pulsatile pump was connected to the vascular model. The catheter was mounted in the vascular model and the proximal wire of the stent was fixed to the gripper. The force generated by pulling the proximal wire of the stent was measured using an automatically motorized stage with a fixed load cell.

**Figure 4:**
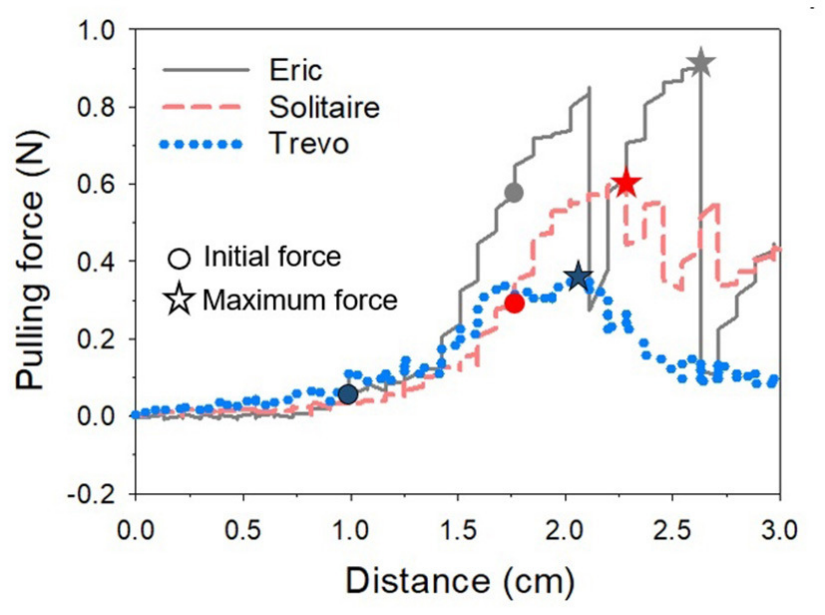
One of several graphs measuring the pulling force according to the movement distance of the motorized stage for each stent. The value when the stent starts to move is regarded as the initial force, and the highest value is considered as the maximum force while the stent is moving. The graph shows that Eric's pulling force is the highest and fluctuates widely. *Indicates the maximum force of each stent.

In order to use the created model in the experiment, it was cut leaving only the ICA, proximal M2, and proximal A2 regions. The mean internal diameter of the vascular model was 5 mm for ICA, 2.7 mm for M1, and 2.5 mm for M2. The frictional forces of Trevo XP (4 x 20 mm, Stryker, Kalamazoo, Michigan, USA), Solitaire 2 (4 x 20 mm, Medtronic, Irvine, California, USA) and Eric 4 (4 x 18 mm, Microvention, Aliso Viejo, California, USA)were analyzed in this experiment (Figure 2). All stents were advanced to the M1 segment of the vascular model before retrieval. After full expansion of the stent in the M1 segment, a motorized traction device retrieved the stent. A load cell (1 kg, BONGSHIN, Inc.) and digital indicator (BS-205, BONGSHIN, Inc.) were used to measure the friction force. The load cell was fixed on a motorized stage. The pulling load was measured using a data acquisition board (USB-6009, National Instruments, Inc.). As the motorized stage moved in a proximal direction with a velocity of 1 mm/s, the stent retriever connected to the load cell was withdrawn. The friction force was measured from the time the motorized stage began to move until the stent exited the internal carotid artery. The inside of the silicon vascular model was filled with saline solution, and a pulsatile blood pump (Model 1405 PBP, Harvard Apparatus, Inc.) was used to generate a pulsating flow with approximately physiological conditions (5 ml stroke volume; 60/min stroke rate) (Figure 3). Two forces were measured in this experimental study. The first was the initial force, which was defined as the force at which the stent begins to move within the artificial silicon vessel. The second was the maximum force, which was defined as the force at the point of maximum resistance during the entire retrieval process (Figure 4). The total pulling energy generated while the motorized stage moved 3 cm was calculated using the measured area of each graph. Retrieval attempts were repeated and measured 10 times for each stent device.

### Statistical analysis

Frictional resistance values for each stent were presented as mean ± SD. Frictional resistance values were compared and analyzed using the Dunnett T3 test. Results were considered statistically significant for *P* < 0.05.

## Results

**Figure 5:**
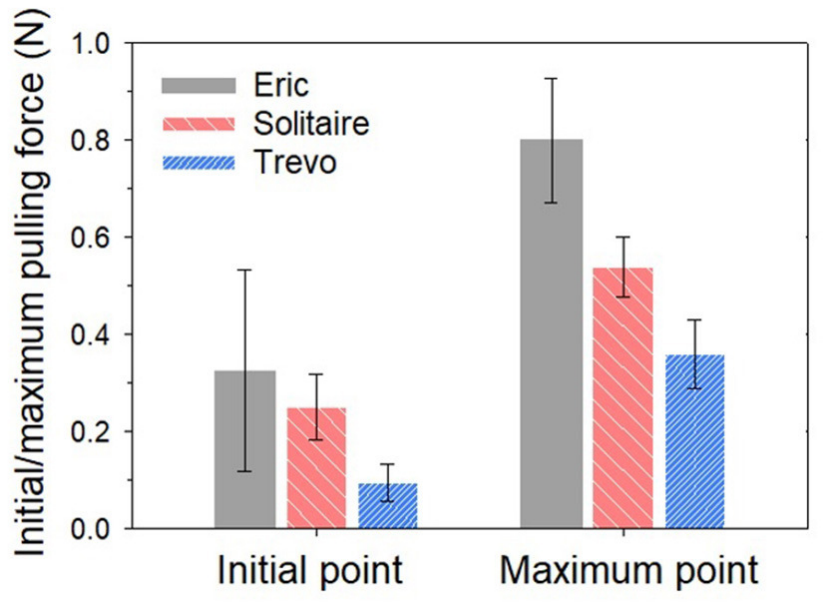
Initial and maximum pulling force comparison for three stents.

**Figure 6:**
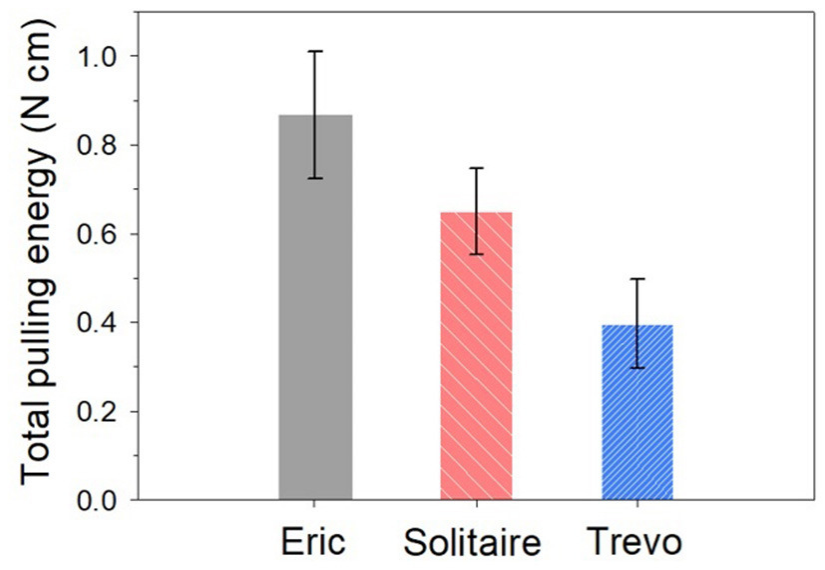
Total pulling energy comparison for three stents.

Table 1 showed the results of the force retrieval experiment. For both the initial and maximal retrieval forces, Trevo demonstrated the lowest resistance values and Eric demonstrated the highest resistance values. Results for the initial retrieval force were as follows: Trevo, 0.09 ± 0.04 N; Solitaire, 0.25 ± 0.07 N; and Eric, 0.33 ± 0.21 N. Results for the maximal retrieval force were as follows: Trevo, 0.36 ± 0.07 N; Solitaire, 0.54 ± 0.06 N; and Eric, 0.80 ± 0.13 N (Figure 5). The maximum force did not occur at the beginning of the retrieving process for any device. Results for total pulling energy (N·cm) were 0.40 ± 0.10 in Trevo, 0.65 ± 0.10 in Solitaire, and 0.87 ± 0.14 in Eric (Figure 6).

**Table 1:** Retrieval frictional forces associated with three stent retriever devices.

**Stent**	Initial force (*N*)^*^	Maximum force (*N*)^**^	Total pulling energy (N·cm)^**^
Trevo	0.09 ± 0.04	0.36 ± 0.07	0.40 ± 0.10
Solitaire	0.25 ± 0.07	0.54 ± 0.06	0.65 ± 0.10
Eric	0.33 ± 0.21	0.80 ± 0.13	0.87 ± 0.14

* By the Dunnett T3 test, P < 0.001 in Trevo – Solitaire, P = 0.035 in Trevo – Eric, P = 0.699 in Solitaire – Eric.

** P < 0.05 in all comparisons between stents.

Differences between the stent retrievers were also determined. For initial resistance, the differences were as follows: Solitaire – Trevo, 0.1583, *p* < 0.001; Solitaire – Eric, −0.0756, *p* = 0.699; Trevo – Eric, −0.2338, *p* = 0.035. For maximal resistance, the differences were as follows: Solitaire – Trevo, 0.1889, *p* < 0.001; Solitaire – Eric, −0.2615, *p* = 0.001; Trevo – Eric, −0.4505, *p* < 0.001. For total pulling energy, the differences were as follows: Solitaire – Trevo, 0.2485, *p* = 0.001; Solitaire – Eric, −0.2218, *p* = 0.001; Trevo – Eric, −0.4703, p=0.001. Significant differences were shown for all comparison values except for the initial force between Solitaire and Eric. The greatest differences in both initial and maximum forces were between the Trevo and Eric devices.

## Discussion

This is the first study to our knowledge to investigate frictional forces of SRT devices in a realistic vascular model. The main finding of our pilot study was that different stent retriever devices have statistically significant differences in associated frictional forces, which could affect different rates of complications in real clinical practice. Future studies using a realistic vascular model are warranted to assess more detailed areas of the vasculature and evaluate any associations between frictional forces using SRT devices and real-world complications.

To date, several studies have analyzed *in vitro* stent retrieval (23–25). However, the artificial vascular models used in these studies were designed to have only simple curvatures that were not similar to real cerebral vascular structures. To determine how stents work in the human cerebral artery, it is necessary to create a more similar vascular environment. Therefore, we attempted to develop a cerebral artery model using 3d printing based on a real human cerebral angiogram. In order to realize elasticity similar to that of an actual blood vessel, silicone elastomer-hydrogel skin multilayers were used (19, 26, 27). In addition, an inner hydrogel coating was used to provide lubrication similar to the endothelium of real human blood vessels (22, 28). During the stent retriever test, blood flow similar to human physiology was also realized using a pulsatile blood pump.

The three devices we investigated regarding their frictional force were the Trevo XP, the Solitaire 2, and the Eric 4. The Trevo XP is a wire-mounted non-detachable stent with a specialized strut form and cut surface that minimizes the effects of radial forces on vascular walls (29). The solitaire stent has a more rigid stent strut, making it more difficult to navigate within vessels (23). This feature could lead to a higher frictional force. Eric is an embolus retriever with an interlinked case and a geometrical design different from other stent retrievers. Gruber et al. reported that intraprocedural complications were generally low for all three stent retrievers (30). Previously, Arai et al. (13) evaluated vascular damage caused by stent retriever thrombectomy devices using a histological examination in an animal model. In that study, the authors compared the Solitaire FR 4 mm and the Trevo ProVue. They identified vascular damage after stent retrieval by observing intimal thickening. They concluded that the Trevo appeared to induce less vascular damage compared to the Solitaire FR. Likewise, differences in intimal thickening between devices evaluated in this study are hypothesized to be due to differences in the structure of the stents. In accordance with that previous study, our experiments showed that the Trevo stent had a statistically lower friction value than Solitaire or Eric.

Similarly, we found Eric had the highest retrieval frictional force in our experimental study. Interestingly, the contact surface of Eric to the vessel wall is smaller than the other retrievers, and this has been offered as an advantage of the device (31, 32). However, in general, the friction force is independent of the contact area. Since the frictional force is proportional to the normal force and the friction coefficient at the contact surface, regardless of the contact area, it may explain why Eric had the highest retrieval frictional force, given it exerted the largest radial force. Machi et al. (23) reported that the radial force of Eric was the highest in comparison to Trevo and Solitaire. Therefore, our experimental results on the difference in pulling force for each stent seem appropriate.

On the other hand, the maximum pulling force during the experiment was not the point at which the stent moved. This result was contrary to common sense that the maximum static friction force is higher than the kinetic friction force. We speculate that the curvature of the vascular model may have influenced the pulling force. The frictional force can change according to the curvature (33). Our vascular model has several curvatures, and frictional force may have changed according to the changed vascular curvature as the stent moved. Since the M1 of our vascular model has a small curvature, we speculate that a higher frictional force can occur compared to the initiation of stent retrieval at the high curvature after passing through the M1. Additional studies should be considered to confirm a clear correlation between the change in pulling force according to the curvature of blood vessels.

Our study has several limitations. First, it was *in vitro* study. We designed an artificial plastic vascular model. Although it was designed based on an actual human vessel, interaction with the inner surface of the model might not be comparable with the human endothelium. Second, it is difficult to identify exactly how the results of this experiment are related to an actual clinical environment. Together with the frictional force of stent retrievers, there are many factors to affect the injury to the surrounding vessel in the real SRT practices, such as vessel diameter, presence of underlying atherosclerotic stenosis, anatomic tortuosity, clot characteristics, and etc. Further studies on artificial vessel models considering those variables seem warranted to overcome these limitations.

## Conclusion

We experimentally found that the retrieving frictional force is different depending on the stent retriever; the Trevo XP stent showed the lowest frictional force during retrieval and the Eric 4 showed the highest. A realistic vascular model was used for this experiment. Although this was a pilot study for this vascular model, we believe this experiment shows the value of such realistic models for future studies.

## Data availability statement

The raw data supporting the conclusions of this article will be made available by the authors, without undue reservation.

## Ethics statement

The studies involving human participants were reviewed and approved by Kyungpook National University Hospital. Written informed consent for participation was not required for this study in accordance with the national legislation and the institutional requirements.

## Author contributions

Conception and design: D-HK, JP, and JK. Conducting experiments: YK, WS, B-JK, MK, S-YY, and JL. Analysis and interpretation of data: JL, YK, and WS. Drafting the article: YK and WS. Statistical analysis: YK. Administrative, technical, and material support: JP. Study supervision: JK and D-HK. All authors contributed to the article and approved the submitted version.

## Funding

This research was supported by a grant from the Korea Health Technology R&D Project through the Korea Health Industry Development Institute (KHIDI) and funded by the Ministry of Health and Welfare, Republic of Korea (grant number: HI15C0001).

## Conflict of interest

The authors declare that the research was conducted in the absence of any commercial or financial relationships that could be construed as a potential conflict of interest.

## Publisher's note

All claims expressed in this article are solely those of the authors and do not necessarily represent those of their affiliated organizations, or those of the publisher, the editors and the reviewers. Any product that may be evaluated in this article, or claim that may be made by its manufacturer, is not guaranteed or endorsed by the publisher.
